# Overconsumption of Energy and Excessive Discretionary Food Intake Inflates Dietary Greenhouse Gas Emissions in Australia

**DOI:** 10.3390/nu8110690

**Published:** 2016-10-31

**Authors:** Gilly A. Hendrie, Danielle Baird, Brad Ridoutt, Michalis Hadjikakou, Manny Noakes

**Affiliations:** 1 CSIRO Health and Biosecurity, P.O. Box 10041, Adelaide 5000, South Australia, Australia; gilly.hendrie@csiro.au (G.A.H.); danielle.baird@csiro.au (D.B.); manny.noakes@csiro.au (M.N.); 2 CSIRO Agriculture, Private Bag 10, Clayton South 3169, Victoria, Australia; 3 Sustainability Assessment Program (SAP), School of Civil and Environmental Engineering, UNSW Australia, Sydney 2052, NSW, Australia; m.hadjikakou@unsw.edu.au

**Keywords:** greenhouse gas emissions, sustainable diet, discretionary foods, Australia, environmental impacts

## Abstract

Population dietary guidelines have started to include information about the environmental impacts of food choices, but more quantifiable evidence is needed, particularly about the impacts associated with discretionary foods. This paper utilised the 2011–2012 Australian Health Survey food intake data along with a highly disaggregated input–output model to estimate the greenhouse gas emissions (GHGe) of Australians’ dietary intake, and compare current patterns of eating which vary in diet quality and GHGe to the recommended diet. The average dietary GHGe were 18.72 ± 12.06 and 13.73 ± 8.72 kg CO_2_e/day for male and female adults, respectively. The correlation between total energy and GHGe was *r* = 0.54 (*p* < 0.001). Core foods contributed 68.4% and discretionary foods 29.4%. Within core foods, fresh meat and alternatives (33.9%) was the greatest contributor. The modelling of current dietary patterns showed the contribution of discretionary foods to GHGe was 121% greater in the average diet and 307% greater in the “lower quality, higher GHGe” diet compared to the recommended diet. Reducing discretionary food intake would allow for small increases in emissions from core foods (in particular vegetables, dairy and grains), thereby providing a nutritional benefit at little environmental expense. Public health messages that promote healthy eating, eating to one’s energy needs and improved diet quality will also contribute to lowering GHGe.

## 1. Introduction

Dietary guidelines issued by governments are mainly focussed on nutrition and health issues related to nutrient adequacy, overconsumption and lifestyle diseases. However, increasingly, consumers and government agencies are interested in the environmental impacts of the food supply, how it is consumed and how to minimise food wastage. This interest has resulted in health and sustainability being integrated into dietary recommendations [1,2,3,4]. The Food and Agriculture Organization of the United Nations has defined sustainable diets as “those with low environmental impacts which contribute to food and nutrition security and to healthy life for present and future generations. Sustainable diets are protective and respectful of biodiversity and ecosystems, culturally acceptable, accessible, economically fair and affordable; nutritionally adequate, safe and healthy; while optimizing natural and human resources” [5].

Internationally, there is the rapid growth in the topic of sustainable diets, mostly focussed on the greenhouse gas emissions (GHGe) associated with dietary intake [6,7,8,9,10,11]. A feature of this literature is the emphasis on the environmental side of the discussion, leading to the exploration of diets with fewer livestock products as these usually feature prominently in the GHGe profile of most diets [10,12,13,14]. However, there are many factors which need to be considered in defining and promoting sustainable diets, foremost the trade-off between environmental responsibility and nutritional adequacy [8]. Approaches which solely target changes in consumption of single food products, such as red meat, have limited use given that populations consume a range of food in differing quantities in a given dietary pattern. In addition, focusing on the environmental benefits of more specialised diets such as vegetarian or vegan patterns of eating is worthwhile on the global scale but may have limited use when trying to develop a science base for population dietary guidelines [15]. Excluding whole food groups can be problematic. For example, animal products are a key source of several micronutrients in the Western diet, which can be challenging to replace when all animal products are excluded.

More recent work examining whole dietary patterns has shown synergies between what is nutritionally and environmentally healthy [1,16,17]. In previous research undertaken in Australia, using intake data from 1995, we found that consuming a diet consistent with the Australian Dietary Guidelines would lower greenhouse gas emissions by 25% relative to the average diet at that time, and also result in fewer discretionary food choices meaning a healthier, and more environmentally friendly, diet overall. Research conducted in the United Kingdom has also shown that changing population food choices to meet their government dietary requirements could help towards mitigating climate change [18], while other European studies have shown the Mediterranean diet is a healthy and sustainable approach to promote [1,19]. However, these findings may be country specific depending on how their population’s current diet compares to guidelines in terms of both diet quality and quantity of food consumed. Another European study has shown changes towards a healthier diet results in minimal environmental benefits [20], or even in the French context, diets which were highest in nutritional quality were not lowest in greenhouse gas emissions [21,22,23]. A recent review of studies linking the nutritional composition of dietary patterns to their greenhouse gas emissions, concluded that some diets that are lower in greenhouse gas emissions are actually higher in sugar and lower in micronutrients [6].

Overconsumption of energy, regardless of the food source of this energy, also relates to the greenhouse gas emission of dietary intake. Vieux et al. (2012) showed a significant and positive relationship between diet-related greenhouse gas emissions and energy intake in a representative sample of French adults [24]. Eating beyond one’s needs contributes to avoidable environmental impacts, and promoting a reduction in energy intake is consistent with messages of healthy eating, healthy weight and obesity prevention.

In Australia, the national dietary guidelines include a brief appendix which addresses environmental issues, highlighting the detrimental impacts of overconsumption, some food production systems and food wastage, as well as the benefits of eating according to the guidelines, choosing seasonal and local products and products with lower environmental impacts [25]. Increasingly countries are also considering the sustainability and implications of their guidelines for healthy diets [1,26,27,28]. While nutritional guidelines are starting to incorporate this environmental perspective, there is still a real need to provide consumers with more scientifically accurate, product and country specific information on the environmental impacts of their food choices and dietary patterns. This involves quantifying the differential impacts between broad core or “healthy” food groups such as meat, dairy, vegetables and grains, as well as considering the environmental impacts of individual food products, including those associated with surplus consumption such as discretionary foods.

The release of the 2011–2013 Australian Health Survey and the recent availability of highly disaggregated greenhouse gas emissions factors for Australia have provided an opportunity to re-examine the greenhouse gas emissions from the contemporary Australian diet. The aim of this paper is to utilise this rich dataset on Australians’ food consumption along with the updated estimates of food sector greenhouse gas emissions to quantify the greenhouse gas emissions of the current Australian daily diet and compare current patterns of eating, which vary in the diet quality and greenhouse gas emissions, to the diet recommended in the population dietary guidelines.

## 2. Materials and Methods

The general approach and principal methodological challenge involved integrating the latest Australian Health Survey data on food consumption of Australian adults with a highly disaggregated input–output model (see Section 2.2) to estimate the greenhouse gas emissions of the Australian populations’ dietary intake. The integrated model was then used to explore the relationship between GHGe and diet quality.

### 2.1. Dietary Intake Data

The 2011–2013 Australian Health Survey (AHS) is the most recent and most comprehensive population health survey conducted in Australian by the Australian Bureau of Statistics (ABS). The National Nutrition and Physical Activity Survey formed part of the AHS and involved collecting detailed dietary intake information from over 12,000 participants (adults and children) across Australia [29]. Dietary intake data were collected using a 24-h recall process, whereby participants recall all foods and beverages consumed on the day prior to the interview. We used one day of dietary recall available from 9341 Australian adults. To ensure the resulting sample was representative of the Australian population, the survey was conducted using a stratified multistage area sample of private dwellings, which means that all sections of the population were represented by the sample. Detailed sampling and demographic information of the sample is available from the Australian Bureau of Statistics [30].

To allow food and nutrient intakes to be estimated from the dietary intake data, the Australian Bureau of Statistics uses a classification system to be able to group similar foods together. This classification system is based on a three-tiered structure. Each unique food item has an 8-digit survey ID assigned. The first two digits of this ID refer to the major food group the food belongs to, based on its key ingredient. There are 24 major food groups, which are then broken down into 132 sub-major food groups, which are further broken down into 500 minor groups [29].

Using this classification system, each individual food (approximately 5500 items including miscellaneous foods and supplements) was assigned an environmental sector code, predominately at the 8 digit level. Single component foods such as chicken was assigned as 100% to the corresponding environmental code, whereas multicomponent foods or mixed dishes, such as chicken and vegetable casserole, were assigned as a proportion of their ingredients up to a maximum of five ingredients. The proportion for these dishes was based on the recipe files provided by the ABS that accompanying the Australian Health Survey [31]. Some foods or dishes for which this detailed disaggregation procedure was carried out include: tea and coffee with varying amount of milk/water; porridge; sandwiches, wraps and hamburgers; mixed dishes where meat was the main ingredient and mixed dishes where a cereal grain was the main ingredient. Table 1 provides some examples of this disaggregation into ingredients. The meat and alternatives (such as beef, lamb, chicken, and egg) and cereal grains (such as bread, pasta, rice, and noodles) source was assigned accordingly for each food.

### 2.2. Greenhouse Gas Emissions Data

The GHGe associated with different food products were estimated using environmentally extended input–output (EEIO) analysis. EEIO is a commonly used method for estimating the full supply chain environmental impacts of food consumption on the basis of household expenditure data, which act as the final demand vector in an environmentally extended Leontief model [20,27,32,33]. The greenhouse gas emissions factors include the total CO_2_ equivalents from all sources encompassing the entire life cycle (including the full life cycle impacts of all upstream inputs) from the point of production to the point of purchase. In this study we employ a highly disaggregated multi-regional supply-use table (SUT) for the Australian economy for 2009 sourced from the Industrial Ecology Virtual Laboratory [34] with greenhouse gas extensions supplied by the National Greenhouse Gas Inventory [35]. The custom-made national SUT extracted for the purposes of this study has 200 economic sectors, including 192 agri-food sectors. The table is based on the official input–output tables published by the Australian Bureau of Statistics (ABS) for 2009/10 [36] which have been disaggregated from 111 sectors into 1284 products based on detailed industry–product relationships [37] and subsequently rebalanced using a constrained optimisation balancing algorithm [34].

Each food or ingredient item in the dietary data classification system was matched as closely as possible to one of the 192 agri-food sector categories. The level of food product disaggregation employed in this study is thus considerably higher compared to previous EEIO studies related to food consumption. Whilst process-based life cycle assessment can offer comparable levels of product detail, it lacks the upstream process completeness of EEIO due to truncation errors arising from the need to define a system boundary. The original carbon dioxide equivalents (CO_2_e) GHGe factors [35] were disaggregated from around 80 sectors to now 200 sectors (192 of which were food-related), on the basis of economic output weights. This has allowed for a more refined matching of raw agricultural or processed food products to the individual foods within the classification system, thereby avoiding the common pitfall of aggregating product data to match the input–output classification which can give rise to errors in the form of aggregation bias [38]. Additional considerations were necessary to calculate the carbon intensity of different fresh meat sectors in order to adequately distinguish between different meat products, following the procedure previously described in Hendrie, Ridoutt, Wiedmann and Noakes [39]. The GHGe intensities per dollar (A$) in basic prices (not including margins) were finally converted to purchasers prices using the latest purchaser to basic price relationships from the ABS input–output tables [36] to ensure compatibility with consumer food expenditure data. Greenhouse gas emissions intensities per dollar are available as Table A1.

### 2.3. Estimation of Dietary Greenhouse Gas Emissions

To integrate the consumption data (in grams) with the greenhouse gas emissions factors (in dollars), grams of food needed to be expressed in dollars using current market price. The input–output tables are in basic prices whereas consumer expenditure and market prices are in purchaser prices (all transportation margins and taxes are included). A conversion is necessary to ensure compatibility. The ABS publishes the ratios necessary to carry out this conversion for each product as part of the input–output table datasets [40]. To convert grams of food consumed to dollars of food consumed (as purchase price) market price per gram for each food item was assigned using a contemporary Australian supermarket pricing database [41]. The median price for a food category was calculated and assigned to food items using a similar approach to the ingredients described above. Single food items were assigned a 100% of the price of that ingredient, whereas multicomponent foods or mixed dishes were assigned a total price in the same proportion as the ingredients.

To convert between raw and cooked products in the input–output model and food consumption data, adjustment factors were applied. Cooked meat to raw meat was multiplied by a factor of 1.4 and cooked rice and pasta back to the raw ingredient by dividing by a factor of 3. A total greenhouse gas emission factor per food item as consumed was calculated as the adjusted grams consumed × price per gram × greenhouse gas emissions per dollar.

### 2.4. Statistical Analysis

Intake of individual foods and components from mixed dishes were aggregated up for the day and total food group intake (serves), energy (kilojoules) and associated greenhouse gas emissions for each individual on the day they were surveyed were estimated. Population means and standard deviations were estimated and all data weighted using the person weighting factor provided by the ABS. The food groups of interest were the five core food groups and discretionary foods as described in the Australian Dietary Guidelines. Core food groups include fruit, vegetables, dairy and alternatives, meat and alternatives, breads cereal and grain foods, as well as unsaturated fats and oils. Within meat and alternatives, we examined red meat (beef, lamb and pork), poultry, fish, vegetarian alternatives (tofu, eggs, and nuts) and other native meats and offal. Sub groups within discretionary foods were also examined including processed meat and meat dishes, alcoholic beverages, sugar sweetened beverages, dairy desserts, savoury and sweet biscuits, sweet and savoury pies, muesli bars and confectionary, and fried potatoes.

To examine the relationship between overconsumption of energy and dietary greenhouse gas emissions, individuals’ minimum energy requirement calculated as their basal metabolic rate (based on their age, gender and weight) was compared to their reported energy intake (as a proportion, i.e., intake divided by BMR), where the higher the number the greater the likelihood of overconsumption of energy relative to requirement.

### 2.5. Modelling of Dietary Pattern Scenarios

Individuals were sorted into four quadrants, ranking them as either higher or lower diet quality, and higher or lower dietary GHGe in comparison to the mean (and removed those individuals who were within 0.25 standard deviations of the mean for both diet quality and GHGe). Here, diet quality was estimated using the Dietary Guideline Index which reflects overall compliance with the Australian Dietary Guidelines in terms of the amount and quality of food consumed from the core food groups, discretionary foods and beverages, as well as diet variety [42]. The index is comprised of 11 components and individuals receive a diet quality score out of 100, where a higher score reflects greater compliance with the Guidelines.

In this analysis, we have modelled the greenhouse gas emissions from three current eating patterns and compared these to the recommended dietary intake pattern from the Australian Guide to Healthy Eating (AGHE). In summary:

(1) Current pattern 1: Best of existing Australian adults’ intake “higher quality, lower GHGe”. This group as a whole had an average daily diet quality score of 59 out of 100, and average GHGe of 8.5 kg CO_2_e/day.
(2) Current pattern 2: Worst of the existing Australian adults’ intake “lower quality, higher GHGe”. This group as a whole had an average daily diet quality score of 27 out of 100, and average GHGe of 26.3 kg CO_2_e/day.
(3) Current pattern 3: The average existing Australian adults’ intake. This group as a whole had an average daily diet quality score of 43 out of 100, and average GHGe of 15.3 kg CO_2_e/day.
(4) Recommended pattern: The recommended dietary intake pattern as per AGHE. This dietary pattern would receive a diet quality score of 100 out of 100. The AGHE recommends number of serves of each core food group and emphasises choosing a variety of foods, but does not prescribe specific foods. For example, in the case of vegetables, it is recommended to eat a variety of types and colours. Greenhouse gas emissions will vary for different age and gender groups depending of serves of food recommended (see Table 3 for recommended serves and GHGe for males and females 19–50 years).

## 3. Results

### 3.1. Total Dietary Greenhouse Gas Emissions and Energy Intake

**Figure 1:**
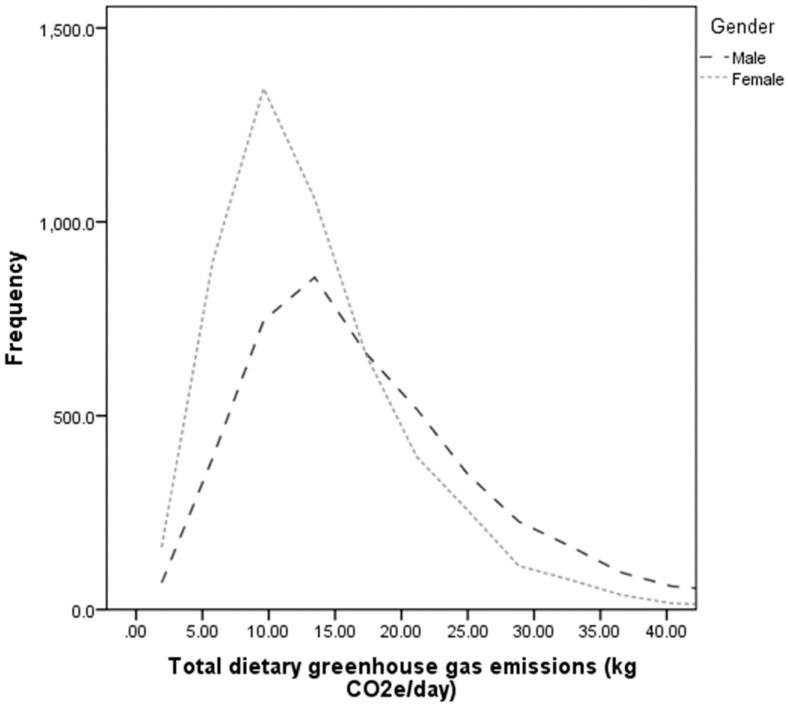
Distribution of total dietary greenhouse gas emissions for Australian males (black dashed line, *n =* 4282) and Australian females (grey dotted line, *n =* 5059).

**Figure 2:**
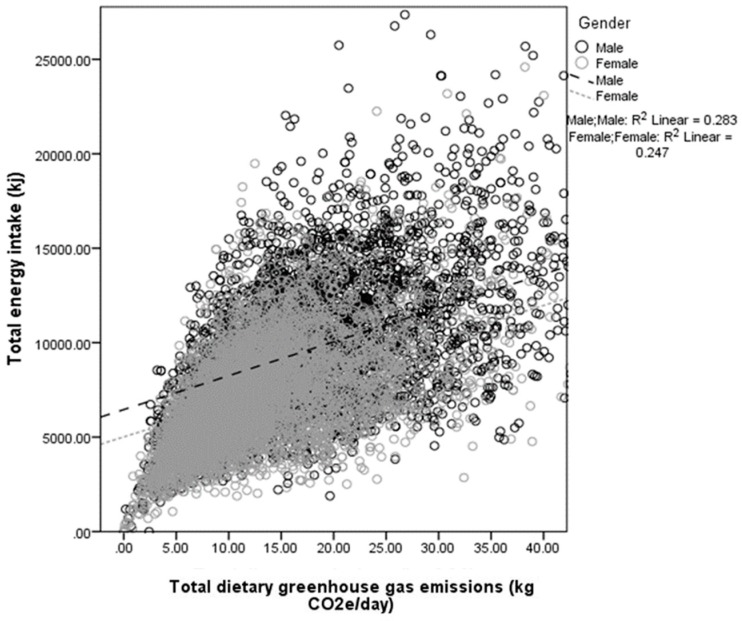
Scatterplot of total dietary greenhouse gas emissions against total energy intake for Australian males (*n =* 4282) (black) and Australian females (grey *n =* 5059).

Figure 1 shows the distribution of total dietary GHGe for adult males and females. The average ± standard deviation GHGe and energy intake for males was 18.72 ± 12.06 kg CO_2_e/day and 9954 ± 3912 kJ, and for females 13.73 ± 8.72 kg CO_2_e/day and 7420 ± 2940 kJ. There was a significant positive correlation between total energy consumed and total dietary GHGe (Figure 2). The Pearson correlation coefficient was *r =* 0.54 (*p <* 0.001) for adults overall (males *r =* 0.52, females *r =* 0.47, *p <* 0.001). The correlation between amount of food consumed (total food in grams) and dietary GHGe was weaker but significant (males *r =* 0.36, females *r =* 0.30, overall *r =* 0.37, all *p <* 0.001). The variation around the line of best fit (indicated by the *R*^2^ value) is also greater when total grams of food is plotted against GHGe (*R*^2^: Males = 0.133, Females 0.093), than for energy against GHGe (Males *R*^2^ = 0.283, Females *R*^2^ = 0.247, Figure 2). There was also a significant and positive relationship between total dietary GHGe and overconsumption of energy in the sample as a whole sample (*r =* 0.48, *p <* 0.001), as well as in males and females separately (males *r =* 0.48, females *r =* 0.46, *p <* 0.001).

### 3.2. Contributions of Food Groups to Total Dietary Greenhouse Gas Emissions

**Figure 3:**
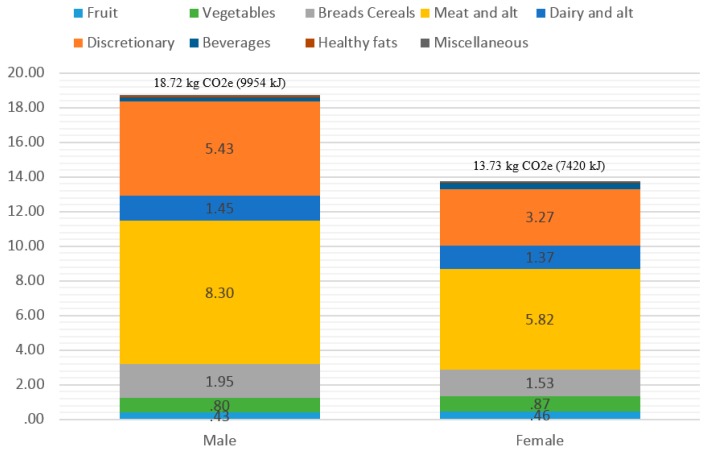
Cumulative average contribution of food groups to total dietary greenhouse gas emissions for Australian males and females.

Figure 3 shows the cumulative contribution of each food group to total dietary GHGe for males and females and Table 2 shows each food group’s contribution as a percentage of total dietary GHGe. The standard deviation is also shown in Table 2 to highlight the large variability in GHGe between individual diets. Despite females having a lower dietary GHGe, the relative distribution of food groups to total GHGe is similar between males and females. Core foods contributed 68.4% to total GHGe and discretionary foods 29.4%. Of the core food groups, fruit (3.5%) and vegetables (6.5%) were the two smallest contributors to total dietary GHGe, and fresh meat and alternatives (33.9%) and discretionary foods (29.4%) the two highest contributors. Within the fresh meat and alternatives food group, red meat contributed 4.86 kg CO_2_e/day (18.8% of total GHGe) and poultry 2.24 kg CO_2_e/day (10.9%) to a total of 18.72 kg CO_2_e/day for males and for females the contributions were 3.20 kg CO_2_e/day (16.5%) and 1.61 kg CO_2_e/day (11.0%) from a total of 13.73 kg CO_2_e/day, respectively. From the subcategories within discretionary foods, processed meats (including dishes containing meat such as burgers, tacos and pizza) and alcoholic beverages were the two highest contributors, contributing 11.3% and 5.7% to total GHGe, respectively (Table 2).

### 3.3. Comparing the Food Intake Adequacy and Greenhouse Gas Emissions from Three Current Eating Patterns to the Recommended Eating Pattern for Adults Aged 19–50 Years

Table 3 shows the average intake (in serves) of each of the core food groups and discretionary foods and GHGe from the four dietary patterns modelled, as an average of males and females aged 19–50 years. The intake of vegetables, grains and dairy foods were below the recommended intakes for all three patterns, and intake of discretionary foods exceeded the recommendation by double for the current average pattern and by four times for the lower quality, high GHGe dietary pattern. Current intakes of meat and alternatives were similar to recommendations (+0.1 serves), but 1.0 serve less than the recommendation in the higher quality, lower GHGe dietary patterns (Table 3).

The total dietary GHGe were highest in the lower quality, higher GHGe diet (25.2 kg CO_2_e/day), followed by the recommended (20.4 kg CO_2_e/day) and current average (19.7 kg CO_2_e/day), and then the higher quality, lower GHGe (13.9 kg CO_2_e/day) dietary pattern.

**Figure 4:**
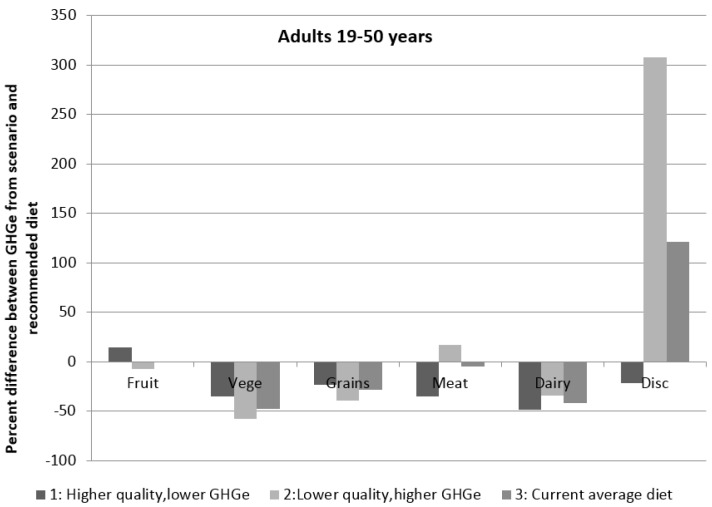
Difference between greenhouse gas emissions from the current average dietary patterns and the recommended daily diet as a percentage of the recommended intake for adults aged 19–50 years.

Figure 4 shows the difference in GHGe between the three current dietary patterns and the recommended dietary pattern as a percentage difference of the recommended intake. The contribution of core food groups to total dietary GHGe was generally lower in the current dietary pattern scenario than the recommended diet, except for contribution from fruit, which was similar. This percentage difference was greatest for vegetables and dairy foods, but was still within 50% for all core food groups. This was overshadowed by the large difference in GHGe from discretionary foods in the current dietary pattern as well as the lower quality, higher GHGe pattern. The contribution of emissions from discretionary foods was 121% greater in the current dietary pattern compared to the recommended dietary pattern, and 307% greater in the lower quality, higher emissions dietary pattern compared to the recommended pattern (Figure 4). The estimated average energy intake from the current average dietary pattern was similar to the recommended dietary pattern, whereas the higher quality, lower GHGe dietary pattern contained approximately 29% fewer kilojoules, and the lower quality, higher GHGe dietary pattern 24% more kilojoules than the recommended diet. It should be noted that there was no adjustment for underreporting in the three current dietary patterns modelled, so true energy consumed is likely to be higher than estimated here (Table 3).

## 4. Discussion

This study has augmented the most recent data on the food intake of Australian adults with the updated environmentally extended input–output model to provide improved estimates of the greenhouse gas emissions of the Australian diet. The updated model provides more than twice as many agri-food sectors as previously published [39], thereby allowing greater sensitivity in the assignment of emission factors to individual foods consumed and deeper understanding of the percentage contribution of different discretionary food products. Our results provide quantitative evidence to support the current dietary guidelines as a way of achieving adequate nutrition and lower overall greenhouse gas emissions, with overconsumption of kilojoules and excessive consumption of discretionary foods being the key drivers of avoidable dietary related greenhouse gas emissions. Of the core foods, fresh meat made the greatest contribution to total dietary emissions, but with greater disaggregation of foods and food sectors, this contribution was found to be more moderate than previously estimated [39].

In shifting the average Australian’s diet towards a nutritionally complete dietary pattern based on the Australian Dietary Guidelines, the contributions from some core food groups would actually need to increase (e.g., vegetables, dairy and grains), some require little change (e.g., fruit and meat) and others would need to decrease (e.g., discretionary foods). The large potential saving in dietary emissions associated with significantly reducing discretionary food intake would allow for the small increases in emissions from core foods, thereby providing considerable nutritional benefit at little or no environmental expense. Similar results have been reported in the Netherlands, where the intake of meat products and extra sweets and snacks needed to decrease to allow for an increase in fruit and vegetables, and a move from the current Dutch diet to their recommended diet would provide an 11% reduction in overall dietary emissions [1].

In the Australian context, fresh meat contributed 34% to total dietary emissions, of which red meat contributed 17.6% and chicken 11%. Another Australian study examining the environmental impacts of the average weekly household food consumption also suggests meat is among the highest contributors to emissions, accounting for about 17% of emissions, however this study only considered CO_2_ and not the other greenhouse gases [33]. Similar results have also been reported internationally. For example, in a Dutch diet where meat products contributed 32% of total dietary CO_2_e [1], and in a French study where meat and deli meat contributed 27% of GHGe [24]. The contribution of meat to dietary emissions can vary internationally as a consequence of differences in intake, country specific differences in emissions from the beef sector, and the categorisation of fresh meat and processed meat. Some studies combine meat and processed meat, however due to the health implications of these different types of meat and the categorisation of processed meat as a discretionary food in the Australian dietary guidelines, we felt it was important to analyse these separately.

Vegetarian and vegan diets exclude meat and/or all animal products and have thereby been shown to have lower dietary emissions. A British study found that compared to the average UK diet, a vegetarian diet reduced greenhouse gas emissions by 22% and a vegan diet by 26%, based on supply data rather than consumption data [13]. However, a recent review article suggests this reduction could be as high as 35% for vegetarian and 55% for vegan diets [10]. There is no doubt vegetarian and vegan diets can achieve very low GHGe, however they need to be adopted with care as they may place individuals at risk of nutrient deficiency [1,43]. This risk extends beyond the example of red meat being a primary source of iron and zinc. In the vegan dietary pattern, the reduction in emissions is partly explained by a lower consumption of dairy products [1], but in Australia most adults are not consuming enough dairy so any advice which further decreases consumption could have implications on their calcium intake and bone health [29].

There were challenges in combining the food classification system used in the National Nutrition Survey with the input–output food sectors, however the addition of over 100 new food sectors has allowed for greater disaggregation of the food supply and composite dishes to produce more accurate greenhouse gas estimates. We have also used a more standardised approach to pricing foods than our previous approach [39] which is an additional strength of the current analysis, and will allow this method to be repeated should another National Nutrition survey be conducted, or even applied to data from other dietary assessment methods. The other strength of this study, and where it is different from some other studies, is that we have modelled three current, and hence credible, dietary scenarios and compared these to current recommendations. By modelling different ways Australians are actually consuming foods, we know any recommendations based on these will be achievable. Small changes may be more realistic for sub-groups of the population, for example encouraging those consuming the lower quality, higher emissions diet to move towards the average daily diet would reduce dietary emissions by between 15%–30% in males and females, and would reduce discretionary food intake by five serves, which provides a health benefit. When theoretical, low emission diets are modelled at the population level, they frequently present nutritional challenges for specific age and gender cohorts and may not be realistic and/or acceptable to many individuals, especially where these aim to reduce emissions by more than 30% [44]. In addition, most studies examining sustainable diets overlook the interrelated problems of excessive energy intake, excessive consumption of energy-dense, nutrient poor non-core foods, and overall nutrient deficiency which characterize many diets. The consistency in the messaging from a health and environmental perspective is encouraging for public health nutrition professionals who are often required to address such concerns from the public.

The greatest body of literature has focused on greenhouse gas emissions associated with dietary intake, and this study has further contributed to this scientific evidence base. However, there are other environmental impacts of the food system which should be considered including impacts related to land and water use, and indirect land use change. In addition, most previous studies are process-based, and have more restricted system boundaries, whereas the input–output life cycle approach used in this study captures all upstream processes, which implies that the numbers will be higher as the scope is more compete (to the point of purchase). This avoids the truncation errors associated with the application of a system boundary in process lifecycle assessment which can be as high as 50% [45,46,47]. Process LCA is commonly used for examining specific supply chains, and estimates of diet-related GHGe using this method tend to be lower [13]. For this reason, the absolute GHGe reported in this study may not be directly comparable to results from other studies where a process LCA approach has been used.

In addition, some assumptions were made which need to be considered. For example, we used a standard price database which assumes that the prices of food products used were representative for all people within the sample. Secondly, we did not adjust for underreporting, however we need to acknowledge that, as is commonly the case in self-reported dietary databases, some degree of misreporting is present in these data. It has been estimated that the average reported energy intake would need to increase by 17%–22% for males and females to achieve energy balance for an average person [29], therefore the estimates of energy intake reported here may be lower than true consumption. In addition, outside the scope of this study was performing a detailed analysis of greenhouse gas emissions within a food group. Emissions from varieties of food within a food group may vary, therefore there may be potential to lower emissions by favouring products with relatively lower emissions. This warrants further investigation. While consumer interest in “sustainable eating” gathers momentum, behaviour change to reduce diet related greenhouse gas emissions may be difficult to implement as consumers have little understanding of which products within a food group have lower emissions and carbon footprint labelling is almost non-existent in the Australian food system.

The calculation of carbon and other environmental footprints associated with dietary change and specific food products is still a work in progress with recent international studies highlighting the great diversity in results due to the use of different functional units and system boundaries [10,48]. According to a recent study of carbon footprints in Australian cities, the total per capita footprint for someone living in one of the main cities in Australia is around 15–25 t CO_2_e per capita [49]. This corresponds to 41–68 kg CO_2_e_e_/capita per day. This implies that our calculated average figure of 18.72 and 13.73 kg CO_2_e/day for male and female adults respectively accounts for a similar percentage of overall emissions as reported elsewhere [50]. Additionally, the food-specific per capita footprint results in this study are fully consistent with previously published values for Australia [33,39,51].

## 5. Conclusions

Some diets that are lower in greenhouse gas emissions are actually less healthy, namely higher in sugar and lower in micronutrients [6] but this study presents quantifiable evidence that eating towards the Australian Dietary Guidelines is healthier based on actual daily food consumption data from a large sample of individuals. This study demonstrates a considerable degree of overlap in the messages of healthy eating and lower GHGe, so a consistent public health message could be developed and promoted relatively easy. Food demand in Australia is steadily increasing with the rising population. Globally, it is expected that food demand will potentially double by 2050 [52]. There is, therefore, a sense of urgency to secure a healthy food supply for future generations, and the findings of this study suggest the most effective strategy to do this is to focus on diet quantity, in terms of eating to one’s energy needs, and diet quality, that is consuming adequate core foods and less discretionary foods. Building and constantly updating a solid scientific evidence base will inform the integration of health and environmental policies and guide behaviour change to reduce greenhouse gas emissions associated with population food choices and nutritional requirements.

## Figures and Tables

**Table 1:** Examples of ingredient composition percentages of mixed dishes.

	Ingredient Number				
	1 (%) *	2 (%)	3 (%)	4 (%)	5 (%)
**Sandwiches, hamburgers, wraps**					
Grain + Meat (or meat alternative) (e.g., cheese burger)	Grain (50)	Meat alt (40)	Sauce (5)	Cheese (5)	-
Grain + Meat (or meat alternative) + Salad (e.g., chicken and salad wrap)	Grain (30)	Meat alt (30)	Veg (30)	Sauce (5)	Cheese (5)
**Mixed dishes where cereal grain main ingredient**					
Grain + Meat (or meat alternative)	Grain (65)	Meat alt (25)	Sauce (5)	Oil (5)	-
**Mixed dishes where Meat (or meat alternative) main ingredient**					
Meat (or meat alternative) + sauce (e.g., meat curry sauce)	Meat alt (50)	Veg (15)	Sauce (5)	Oil (5)	-
Meat (or meat alternative) + Grain + Vege (e.g., meat, vegetable stir fry with rice)	Meat alt (30)	Grain (40)	Veg (20)	Sauce (10)	-

* Percentage cereal grain component (Grain) e.g., bread, pasta, rice, and noodles; Meat and alternatives (Meat alt) e.g., beef, chicken, eggs, and tofu; and Vegetables (Veg) e.g., cooked, raw and salad vegetables within dishes.

**Table 2:** Average percentage contribution of food groups to total dietary greenhouse gas emissions for Australian males and females.

	Male (*n =* 4282)		Female (*n =* 5059)		Total (*n =* 9341)	
	Mean	SD	Mean	SD	Mean	SD
Fruit	2.9	5.0	4.0	6.6	3.5	5.9
Vegetables	5.5	9.5	7.5	11.0	6.5	10.3
Breads and cereals	13.5	16.0	13.7	16.2	13.6	16.1
Fresh meat and alternatives	34.7	29.7	33.1	29.5	33.9	29.6
*Red meat*	*18.8*	*27.5*	*16.5*	*26.9*	*17.6*	*27.2*
*Poultry*	*10.9*	*20.3*	*11.0*	*20.5*	*11.0*	*20.4*
*Fish*	*3.1*	*10.0*	*3.4*	*10.7*	*3.3*	*10.4*
*Vegetarian alt*	*1.9*	*7.6*	*2.1*	*8.7*	*2.0*	*8.2*
*Other: Reptiles, offal*	*0.1*	*1.0*	*0.0*	*0.6*	*0.0*	*0.8*
Dairy	9.4	11.3	11.5	12.7	10.5	12.1
Discretionary foods	31.8	25.7	27.0	23.8	29.4	24.8
*Processed meat, burgers, tacos, pizza*	*12.6*	*19.5*	*10.0*	*17.8*	*11.3*	*18.7*
*Alcoholic beverages*	*7.0*	*12.1*	*4.4*	*10.3*	*5.7*	*11.3*
*Sugar sweetened beverages*	*3.4*	*5.9*	*2.8*	*6.0*	*3.1*	*5.9*
*Dairy based desserts, cream, butter*	*2.1*	*5.5*	*2.0*	*5.5*	*2.1*	*5.5*
*Savoury and sweet biscuits, cakes*	*1.9*	*4.1*	*2.4*	*4.7*	*2.2*	*4.5*
*Sweet and savoury pastries and pies*	*1.5*	*4.9*	*1.6*	*5.8*	*1.5*	*5.4*
*Muesli bars, confectionary and choc*	*1.5*	*3.1*	*1.9*	*4.0*	*1.7*	*3.6*
*Other—stock, salt, dry soups*	*1.1*	*3.8*	*1.4*	*4.1*	*1.2*	*3.9*
*Fried potato and extruded snacks*	*0.7*	*2.2*	*0.6*	*2.3*	*0.6*	*2.3*
Other beverages (non sugar sweetened)	1.6	5.1	2.4	7.2	2.0	6.2
Healthy fats and oils	0.4	1.4	0.6	1.8	0.5	1.6
Miscellaneous	0.3	3.0	0.2	1.2	0.2	2.3

*Italics* indicates sub-groups within the major food group.

**Table 3:** The average food intake (in serves) and dietary emissions from the three current dietary pattern scenarios and the recommended dietary pattern, for adults aged 19–50 years *.

Adults 19–50 years	Higher Quality, Lower GHGe (7508 kJ)		Lower Quality, Higher GHGe (13,195 kJ)		Current Average (10,224 kJ)		Recommended (10,598 kJ)	
	Intake	GHGe	Intake	GHGe	Intake	GHGe	Intake	GHGe
	(Serves)	(kg CO_2_e)	(serves)	(kg CO_2_e)	(Serves)	(kg CO_2_e)	(Serves)	(kg CO_2_e)
Fruit	2.3	0.8	1.9	0.7	2.0	0.7	2.0	0.7
Vege	3.5	1.3	2.3	0.9	3.0	1.1	5.5	2.0
Grains	4.5	2.2	3.6	1.7	4.2	2.0	6.0	2.8
Meat and alternatives	1.8	6.5	3.2	11.5	2.7	9.5	2.8	9.9
Dairy	1.3	1.5	1.6	1.9	1.5	1.7	2.5	2.9
Discretionary	2.2	1.7	11.2	8.6	6.2	4.7	2.8	2.1
Total GHGe		13.9		25.2		19.7		20.4

* The estimation of energy in the current dietary patterns does not account for underreporting.

## Appendix

**Table A1:** Greenhouse gas emissions per purchase price (kg CO_2_eper Australian dollar (($A) for all food products used in this analysis, categorized into their respective food group).

Food Group	Food Products	GHGe/Purchase Price (kg Co_2_e/$A)
**Fruit (fresh and processed)**	Dried fruit (excl sun-dried)	0.48338
	Kiwi fruit	0.47974
	Fruit juices, single strength or concentrated	0.44525
	Preserved fruit and fruit products	0.43979
	Berries nec—fresh and sun-dried	0.40945
	Grapes sun-dried or for drying	0.39999
	Pears and quinces—fresh and sun-dried	0.39512
	Olives—fresh and sun-dried	0.38884
	Grapes—table	0.37619
	Strawberries	0.36613
	Orchard fruit nec—fresh and sun-dried	0.34844
	Bananas—fresh and sun-dried	0.34317
	Stone fruit—fresh and sun-dried	0.34280
	Citrus fruit—fresh and sun-dried	0.34152
	Apples—fresh and sun-dried	0.33794
	Grapes—wine	0.33149
**Vegetables (fresh and processed)**	Lettuces grown undercover	0.63478
	Fresh vegetable salads, in plastic containers	0.55611
	Mushroom spawn	0.53921
	Vegetable juices (incl mixtures) (incl tomato); mixtures of vegetable and fruit juices	0.53887
	Farmed seaweed	0.53246
	Tomato pulp, puree and paste	0.49218
	Vegetables, prepared or preserved (incl dried or shelled)(excl frozen); pickles and chutney	0.44630
	Vegetables, frozen	0.44326
	Tomatoes grown undercover	0.43371
	Olives—fresh and sun-dried	0.38884
	Other vegetables, fresh or chilled, grown undercover	0.38360
	Beans, french and runner; peas, green or blue grown outdoors	0.35533
	Onions grown outdoors	0.34963
	Carrots grown outdoors	0.33958
	Lettuces grown outdoors	0.33944
	Cabbages, brussels sprouts, cauliflowers and broccoli grown outdoors	0.33546
	Tomatoes grown outdoors	0.32920
	Mushrooms, fresh or chilled	0.32215
	Potatoes, sweet potatoes and edible roots tubers grown outdoors	0.30533
	Other vegetables (incl. melons), fresh or chilled grown outdoors	0.30334
**Breads and cereals**	Cereal grains nec	2.51001
	Oilseeds	2.33390
	Lupins (white or yellow) for grain	1.83608
	Legumes for grain nec	1.73941
	Rice, in the husk	1.59707
	Cereal foods (incl breakfast foods)	0.94889
	Rice (husked, semi-milled or wholly milled)	0.93857
	Rice groats, meals and pellets; other worked cereal grains	0.93798
	Malt (excl malt extract)	0.92090
	Pasta	0.91909
	Wheat and other cereal flours (incl self-raising)	0.91385
	Starch of wheat and corn	0.90673
	Mixes and doughs nec (incl custard powder) for preparation of bakers wares (excl frozen)	0.88976
	Flour mill products nec, for human consumption	0.87563
	Malt extract	0.84356
	Wheat bran for human consumption (excl for breakfast food)	0.82789
	Oats, unmilled	0.67978
	Bread and bread rolls	0.67820
	Grain, sorghum	0.63955
	Barley, unmilled	0.61517
	Wheat (incl spelt) and meslin, unmilled	0.60383
	Dried roots, tubers and vegetables; Flour and meal of vegetables	0.50484
**Fresh red meat**	Beef	3.23960
	Lamb	3.07132
	Pork	1.45418
	Kangaroo	1.26580
**Poultry**	Poultry and poultry products (incl canned)	1.79654
**Fish & seafood**	Rock lobster and crab	0.97399
	Crustaceans, molluscs & aquatic invertebrates nec (chilled, frozen, preserved or otherwise prepared)	0.90976
	Frozen whole fish, fish fillets and fish meat; fish loaf, cake, balls and paste; smoked fish; fish fingers; caviar	0.89430
	Oysters and other aquatic invertebrates nec, live, fresh or chilled	0.88602
	Rock lobster and crayfish (incl tails), chilled or frozen (incl boiled and frozen)	0.88479
	Inedible flours, meals, pellets & other products nec of fish, crustaceans & molluscs or other aquatic invertebrates	0.84087
	Fish, canned	0.80886
	Fish and squid (line fishing)	0.80605
	Coral and similar, shells of molluscs; natural animal sponges; algae, fresh or dried	0.74707
	Freshwater fish and aquatic animals nec	0.74366
	Prawns	0.70514
	Fish (trawling or netting)	0.68971
	Farmed fish and fish hatchery products	0.64590
	Farmed prawns and crustaceans nec	0.50414
	Farmed oysters (including Pearl), paua and molluscs nec	0.39522
**Vegetarian Alternatives**	Eggs	0.82685
	Peanut butter and other nut butters, pastes and purees;	0.60125
	Nuts, roasted	0.58439
	Almonds and macadamias	0.35695
	Edible nuts (excluding Peanuts) nec; Other fruit nec—fresh and sun-dried	0.34630
**Other meats**	Edible offals (excl poultry offals)	1.82509
	Meat (excl fresh) for human consumption	1.73576
	Other animal products nec	1.72872
	Deer	0.65689
	Mixed meat and vegetables, canned	0.60675
**Dairy products**	Cheese and curd	1.30394
	Flavoured whole milk drinks	1.29049
	Sour cream, yoghurt and other cultured milk products	1.28294
	Milk and cream, concentrated or sweetened; lactose and lactose syrup; products of natural milk constituents	1.26744
	Milk based food preparations (excluding malt extracts) and dried milk based mixes	1.26283
	Buttermilk	1.26123
	Processed liquid milk (incl whole milk and skim)	1.19767
Processed meat	Bacon and ham and other dried, salted or smoked pigmeat (incl canned)	1.84050
	Smallgoods nec (incl crumbed lamb cutlets, cured meat (canned or uncanned), frankfurters, saveloys and salami)	1.83591
Alcohol	Beer	0.46729
	Wines (incl sparkling) of grapes and other fruit (excl vermouth)	0.25090
	Spirits and fortified wines	0.23627
	Other alcohol	0.11501
Sugar sweetened beverages	Mineral waters and aerated waters, sweetened or flavoured, bottled	0.48782
	Mineral waters and aerated waters, sweetened or flavoured, canned	0.35982
	Sweetened or flavoured bulk pre-mix & post-mix concentrates for mineral & aerated waters; non-alcoholic beverages	0.35744
	Cordials and syrups; powder flavours for soft drinks; concentrated cordial extracts	0.35320
Dairy based desserts	Ice cream and frozen confections	1.22533
	Cream (incl thickened), not concentrated or sweetened	1.22406
Savoury and sweet biscuits and cakes	Biscuits and biscuit crumbs; rusks; ice cream cones and wafers; unleavened bread	0.65472
	Cakes, pastries and crumpets	0.65356
	Biscuit and bread dough (incl frozen)	0.63559
	Bakers’ wares nec (incl pretzels and frozen pizza) (excl bread and pies)	0.58847
Sweet and savoury pastries and pies	Meat pies	0.62161
	Prepared meals (incl TV dinners), of meat or meat offal	0.61268
Confectionary	Glucose, glucose syrup (incl dextrose) and modified starches (incl dextrins)	0.89764
	Liquid refined sugar, golden syrup, artificial honey, starch and sugar products nec	0.61563
	Crystallised, drained and glace fruit, nuts and peel	0.57841
	Icing sugar, molasses (incl treacle) and sugar nec	0.57003
	Other food preparations containing cocoa (excl chocolate confectionery)	0.53570
	Raw and refined sugar in solid form (incl brown sugar) (excl icing sugar)	0.53402
	Chocolate confectionery (excl chocolate coated biscuits and white chocolate)	0.52437
	Chewing gum, white chocolate and other confectionery not containing cocoa	0.52369
	Cocoa beans (roasted); cocoa paste, powder, butter, fat or oil	0.51854
	Jams	0.48045
Other—stock, salt, dry soups	Edible tallow (excl refined)	1.59973
	Butter	1.25690
	Fats and oils derived from milk (incl butter oil); casein	1.23787
	Refined and processed animal or vegetable oils and fats (incl tallow) (excl neatsfoot, wool grease and lanolin)	1.01827
	Restaurants and catering	0.69551
	Fast food and takeaway	0.64127
	Food products nec (incl jelly crystals, meat pastes)	0.56617
	Pasta products, canned	0.54331
Fried potato and extruded snacks	Corn chips; taco, tortilla and tostada shells	0.56981
	Potato crisps and flakes	0.55735
Beverages	Natural water nec	0.34758
	Natural and artificial mineral waters and aerated waters (excl sweetened or flavoured)	0.23294
	Ice	0.22193
Healthy fats and oils	Crude soya bean, cotton seed, peanut, sunflower, safflower, rape seed, coconut and vegetable oils	1.01000
	Margarine	1.03513
Miscellaneous	Wheat gluten and tapioca	0.90385
	Prepared baking powders	0.86756
	Mustard; worcestershire sauce; mayonnaise and salad dressing	0.58727
	Coffee and tea, including substitutes	0.58032
	Yeast and yeast extracts	0.57738
	Spices	0.57732
	Gelatine	0.57695
	Flavouring essences, industrial	0.56924
	Refined salt (cooking and table)	0.56792
	Soup and homogenised food preparations including fruit, vegetables, meat or composites thereof	0.47864
	Fruit and vegetable based health, invalid or baby preparations	0.47812
	Sauces (excl worcestershire/apple); vinegar (excl wine vinegar)	0.47399
